# Cyclophilin A (CypA) Plays Dual Roles in Regulation of Bone Anabolism and Resorption

**DOI:** 10.1038/srep22378

**Published:** 2016-03-02

**Authors:** Mian Guo, Aaron W. James, Jin Hee Kwak, Jia Shen, Kazunari K. Yokoyama, Kang Ting, Chia B. Soo, Robert H. Chiu

**Affiliations:** 1 Dental and Craniofacial Research Institute and Division of Oral Biology, School of Dentistry, University of California, Los Angeles, CA 90095, USA; 2 Department of Pathology and Laboratory Medicine, David Geffen School of Medicine, University of California, Los Angeles, CA 90095, USA; 3 Dental and Craniofacial Research Institute and Section of Orthodontics, School of Dentistry, University of California, Los Angeles, CA 90095, USA; 4 Department of Orthopedic Surgery, School of Medicine, University of California, Los Angeles, CA 90095, USA; 5 Division of Plastic and Reconstructive Surgery, School of Medicine, University of California, Los Angeles, CA 90095, USA; 6 Graduate Institute of Medicine, Kaohsiung Medical University, Kaohsiung, 80708, Taiwan; 7 Department of Neurosurgery, the Second Affiliated Hospital of Harbin Medical University, Harbin, Heilongjiang, 150086, China; 8 Jonsson Comprehensive Cancer Center and Division of Surgical Oncology, School of Medicine, University of California, Los Angeles, CA 90095, USA

## Abstract

CypA (Cyclophilin A) is a peptidyl-prolyl isomerase previously shown to be required for chondrogenic differentiation and endochondral ossification. However, the effects of CypA on osteoclast activity and bone maintenance are entirely unknown. Here, we show that *Ppia*^−/−^ mice demonstrate low bone mineral density, reduced osteoblast numbers, and increased osteoclast numbers. When isolated from the calvaria, *Ppia*^−/−^ osteoblasts demonstrate decreased osteogenic differentiation, whereas *Ppia*^−/−^ osteoclasts derived from the long bones showed increased osteoclastic activity. Overexpression and gene silencing of CypA verified osteogenic and anti-osteoclastic effects. In osteoblasts, CypA is necessary for BMP-2 (Bone Morphogenetic Protein-2)-induced Smad phosphorylation. In osteoclasts, loss of CypA activates BtK (Bruton’s tyrosine kinase) and subsequently integrates with TRAF6 (TNF receptor-associated factor 6) and/or c-fos signaling to induce NFATc1 (nuclear factors of activated T cells, cytoplasmic 1). Collectively, CypA dually exerts pro-osteogenic and anti-osteoclastic effects. Thus, modulation of CypA may be useful in future efforts targeting osteoporosis.

Osteoporosis is the most common metabolic bone disease, affecting an estimated 10 million Americans^1^. Osteoporosis results from an imbalance between bone formation and resorption. This balance depends on both the number and activity of osteoblastic and osteoclastic cells^2^,^3^,^4^. Numerous pharmaceutical agents have been developed to treat osteoporosis, which are broadly categorized as either anabolic or anti-resorptive. However, new therapies that have both anabolic and anti-osteoclastic effects would represent a major advancement in our ability to effectively treat osteoporotic bone loss.

PPIases (Peptidyl-prolyl isomerases), such as CypA (cyclophilin A), function as novel molecular timers that help control the amplitude and duration of cellular processes^5^. CypA is expressed in both intracellular and extracellular spaces and has important effects in diverse tissue types, including cartilage^6^, skeletal^7^ and cardiac muscle^8^, the vasculature^9^, and the immune system^10^. CypA acts as a signal transducer and activator of transcription via promoting the activation and nuclear translocation of other factors^11^,^12^. For example, CypA is necessary for Stat3 Interleukin-6-induced tyrosine phosphorylation and nuclear translocation in myeloma cell lines^13^. In another example, we previously found that CypA is required for chondrogenic differentiation and endochondral ossification^6^. Moreover, CypA functions as a novel NF-κB (NF-kappaB)/p65 interacting protein^14^ and acts in concert with p65 to mediate BMP-2 (Bone Morphogenetic Protein-2)-induced Sox9 expression^6^. However, the effects of CypA on osteoclast activity and bone maintenance are entirely unknown.

Here, we have identified a novel dual function of intracellular CypA in the regulation of osteoblastic and osteoclastic activity. First, juvenile *Ppia*^−/−^ mice manifested a low bone mineral density (BMD) skeletal phenotype, accompanied by a reduction in the size of the skeleton, reduced osteoblast numbers, and increased osteoclast numbers. *In vitro* studies confirmed that CypA had dual pro-osteogenic, anti-osteoclastic effects. Moreover, CypA appeared to differentially regulate signaling pathways in osteoblasts and osteoclasts. In osteoblasts, CypA was required for BMP-2 induced Smad phosphorylation, while in osteoclasts loss of CypA resulted in BtK (Bruton’s tyrosine kinase) activation and integrated with TRAF6 (TNF receptor-associated factor 6) and/or c-fos signaling to induce NFATc1 (nuclear factors of activated T cells, cytoplasmic 1).

## Results

### High-resolution microCT analysis of Ppia^−/−^ mice

**Figure 1:**
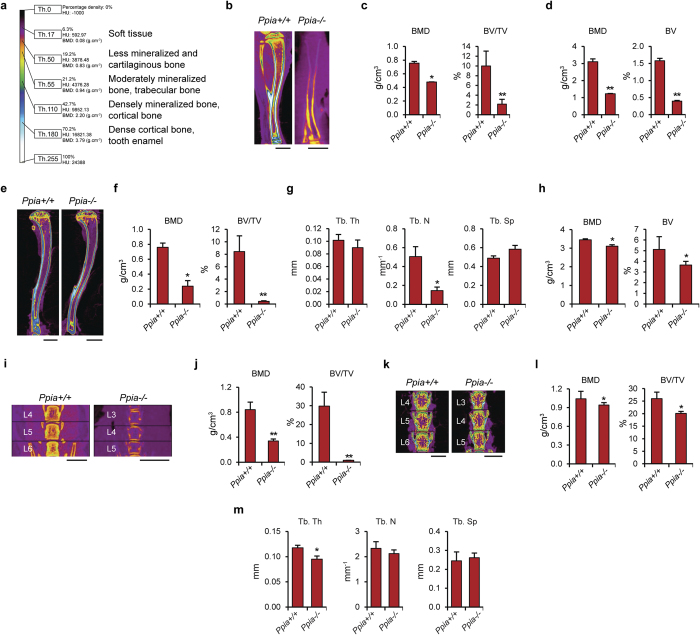
MicroCT analysis of *Ppia*^+/+^ and *Ppia*^−/−^ mice. (**a**) Color scale bar is shown to indicate threshold, HU and BMD values, and tissue types correspondent to select colors. (**b**) Mid-coronal cross sectional images of the tibia from three-week old *Ppia*^+/+^ and *Ppia*^−/−^ mice. Image is color-coded based on tissue density level. *Ppia*^−/−^ tibias showed an average of 26% reduction in length. Scale bars: 2 mm. (**c**) Quantification of trabecular BMD (bone mineral density) and percent bone volume (BV/TV) in the distal tibia. (**d**) Cortical BMD and BV (bone volume) in the tibial shaft. (**e**) Overview mid-coronal cross sectional images of the proximal tibia from 13-week old mice. The *Ppia*^−/−^ tibias showed an average of 11% reduction in length. Scale bars, 2 mm. (**f**) Quantification of trabecular BMD and BV/TV in the proximal tibia. (**g**) Quantification of Tb. Th (trabecular bone thickness), Tb. N (trabecular number) and Tb. Sp (trabecular space) in the proximal tibia, 13 weeks. (**h**) Cortical BMD and BV in the tibial shaft. (**i**) Mid-coronal cross sectional images of the lumbar vertebrae from three-week old *Ppia*^+/+^ and *Ppia*^−/−^ mice. *Ppia*^−/−^ vertebral bodies (at the three terminal lumbar vertebrae analyzed) showed an average of 40% reduction in height with visible reduction in bone quality (volume, density, and structure) compared to WT. Scale bars: 1 mm. (**j**) BMD and BV/TV in the lumbar vertebrae. (**k**) Mid-coronal cross sectional images of the lumbar vertebrae from 13-week old *Ppia*^+/+^ and *Ppia*^−/−^ mice. *Ppia*^−/−^ vertebral bodies (at the three terminal lumbar vertebrae analyzed) showed an average of 13% reduction in height with visible reduction in bone quality (volume, density, and structure) compared to WT. Scale bars: 1 mm. (**l**) BMD and BV/TV in the lumbar vertebrae. (**m**) Quantification of Tb. Th, Tb. N and Tb. Sp in the lumbar vertebrae. n = 6 for WT group at 3 and 13 weeks old. n = 5 for KO group at 3 weeks old. n = 4 for KO group at 13 weeks old. *P < 0.05, **P < 0.01 compared to *Ppia*^+/+^. Data represented as mean and SEM.

The skeletal phenotypes of wildtype (*Ppia*^+/+^) and knockout (*Ppia*^−/−^) mice were analyzed by microCT at three and 13 weeks of age. The overall size of the *Ppia*^−/−^ skeleton was reduced, including a 26% reduction in mean tibia length (Fig. 1a). Next, quantitative differences in trabecular and cortical Bone Mineral Density (BMD), and fractional Bone Volume (BV/TV) were assessed. At three weeks, marked reductions in trabecular bone indices (Fig. 1c) and cortical bone indices (Fig. 1d) were observed. Analysis of the *Ppia*^−/−^ skeleton at 13 weeks of age showed similar findings (Fig. 1e–h).

Next, the lumbar spine of *Ppia*^+/+^ and *Ppia*^−/−^ mice was examined (Fig. 1i–m). The *Ppia*^−/−^ vertebral body showed a 40% reduction in height at three weeks of age, along with a visibly apparent decrease in cortical thickness and mineralization (Fig. 1i). Trabecular bone indices showed a reduction in *Ppia*^−/−^ mice (Fig. 1j). Similar observations were also found in the lumbar vertebrae at 13 weeks of age (Fig. 1k–m), including a reduction in vertebral height (Fig. 1k), and reduction or non-significant trend toward reduction in all trabecular bone indices (Fig. 1l,m).

In summary, *Ppia*^−/−^ mice demonstrate a reduction in measured parameters of trabecular and cortical bone. These findings were consistent across both the appendicular and axial skeleton. Differences were most notable during early skeletal development, but persisted into skeletal maturity.

### Histologic, histomorphometric and immunohistochemical analysis of Ppia^−/−^ mice

**Figure 2:**
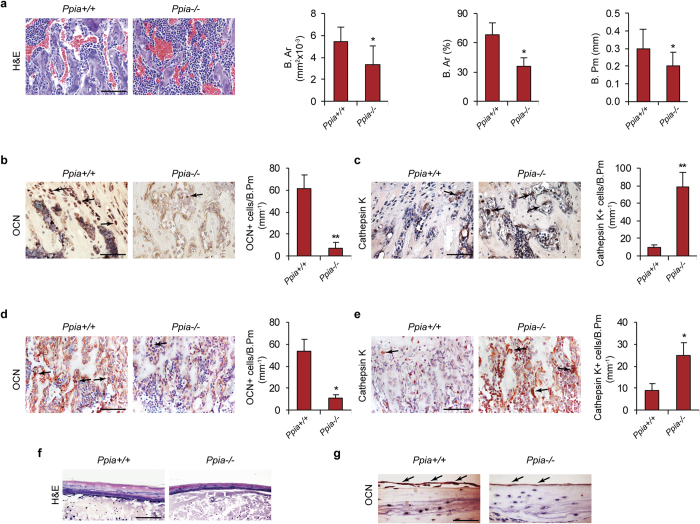
Histologic and immunohistochemical analysis in *Ppia*^+/+^ and *Ppia*^−/−^ mice. (**a**) Representative images of H&E staining of trabecular bone of the proximal tibia in 3-week old *Ppia*^+/+^ and *Ppia*^−/−^ mice. Quantification of B.Ar (Bone Area), percentage B.Ar, and B.Pm (Bone Perimeter). Scale bar: 100 μm. (**b**) OCN (Osteocalcin) and (**c**) Cathepsin K immunohistochemical staining and quantification in 3-week old *Ppia*^+/+^ and *Ppia*^−/−^ mice. Proximal tibia shown. Quantifications expressed as the number of immunoreactive cells per B.Pm. Scale bars: 100 μm. In the case of Cathepsin K staining, only multinucleated bone lining cells were quantified. (**d**) OCN and (**e**) Cathepsin K immunohistochemical staining and quantification in newborn *Ppia*^+/+^ and *Ppia*^−/−^ mice. Proximal tibia shown. Quantifications expressed as the number of immunoreactive cells per B.Pm. Scale bars: 100 μm. **(f,g**) Representative images of the calvaria in 13-week old *Ppia*^+/+^ and *Ppia*^−/−^ mice, including (**f**) H&E and (**g**) OCN immunostaining. Black arrows indicate examples of immunoreactivity. N = 4 in each group. Scale bars: 50 μm. *P < 0.05, **P < 0.01 compared to *Ppia*^+/+^ mice. Data represented as mean and SEM.

Histologic analysis of *Ppia*^−/−^ mice was performed in comparison to wildtype littermates to confirm our radiographic findings. At three weeks of age, a reduction in trabecular bone was observed (Fig. 2a). Histomorphometric analysis confirmed a reduction in B.Ar (Bone Area), percentage B.Ar (% B.Ar), and trabecular B.Pm (bone perimeter) (Fig. 2a). Next, quantification of the numbers of osteoblasts and osteoclasts within the *Ppia*^−/−^ skeleton was performed. Ocn (Osteocalcin) immunohistochemical staining permitted identification of bone-lining osteoblasts, and showed a reduction in osteoblasts per B.Pm (Ocn^+^ cells/B.Pm), Fig. 2b. Next, Cathepsin K immunostaining revealed an increase in Cathepsin K^+^ multinucleated osteoclasts among *Ppia*^−/−^ mice (Fig. 2c). These findings were also confirmed in newborn mice, again showing a reduction in osteoblasts and increase in osteoclasts (Fig. 2d,e). Next, we extended these findings to the cranial skeleton. A reduction in the thickness of the calvarial bone was observed in *Ppia*^−/−^ mice (Fig. 2f), accompanied by a reduction in immunoreactivity for Ocn among periosteal cells and osteocytes (Fig. 2g).

In summary, *Ppia*^−/−^ mice demonstrate a reduction in osteoblast numbers and a converse increase in osteoclast numbers. Moreover, this phenotype was observed across developmental timepoints and locations.

### Effects of CypA dysregulation on osteoblastic and osteoclastic activity

**Figure 3:**
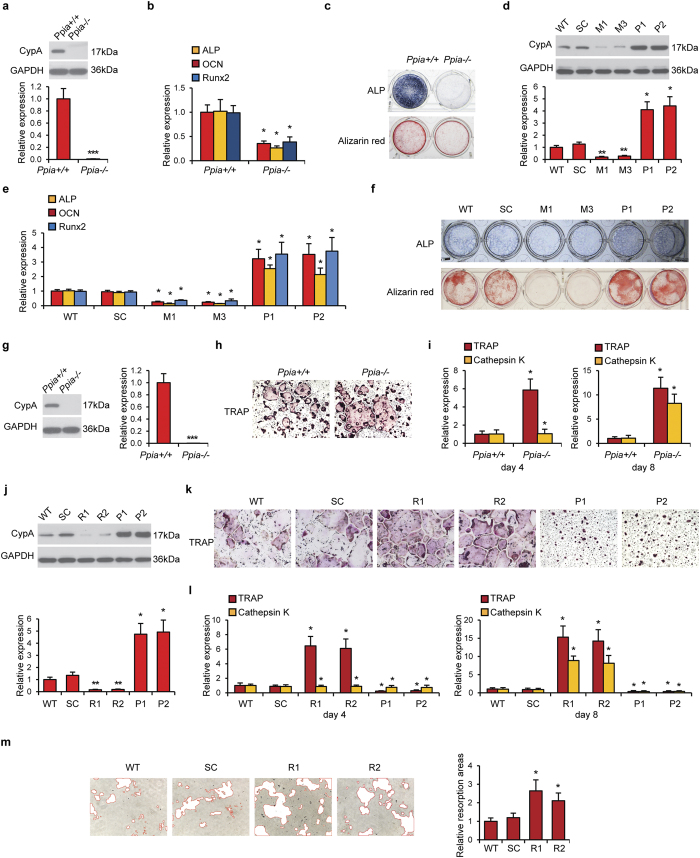
Differentiation of osteoblasts and osteoclasts with CypA dysregulation. (**a**) Western blot of CypA in *Ppia*^+/+^ and *Ppia*^−/−^ primary osteoblastic cells. GAPDH was used as a loading control. (**b**) *ALP, OCN* and *Runx2* expression in *Ppia*^+/+^ and *Ppia*^−/−^ osteoblastic cells after seven days of osteogenic differentiation. (**c**) ALP and Alizarin red staining in *Ppia*^+/+^ and *Ppia*^−/−^ osteoblastic cells after seven and 21 days of osteogenic differentiation, respectively. (**d**) CypA levels assayed in MC3T3-E1 osteoblastic cells after silencing or forced overexpression. Two clones for CypA silencing are referred to as M1 and M3, while two clones for overexpression are designated P1 and P2 (comparison include scrambled control, SC, and wildtype parental cells, WT). (**e**) *ALP, OCN* and *Runx2* expression in WT, SC, M1, M3, P1 and P2 MC3T3-E1 cells after seven days of osteogenic differentiation. (**f**) ALP and Alizarin red staining in WT, SC, M1, M3, P1 and P2 MC3T3-E1 cells during osteogenic differentiation. (**g**) Expression of CypA in *Ppia*^+/+^ and *Ppia*^−/−^ mouse primary osteoclastic cells. (**h**) TRAP staining of *Ppia*^+/+^ and *Ppia*^−/−^ osteoclast cells after seven days of osteoclastic induction. (**i**) *TRAP* and *Cathepsin K* expression in *Ppia*^+/+^ and *Ppia*^−/−^ osteoclastic cells after four and eight days osteoclastic induction. Magnification: 10 ×. (**j**) CypA levels assayed in RAW264.7 osteoclastic cells after silencing or forced overexpression. Two clones for CypA silencing are referred to as R1 and R2, while two clones for overexpression are designated P1 and P2 (comparison include scrambled control, SC, and wildtype parental cells, WT). (**k**) TRAP staining in (**j**). Magnification: 10×. (**l**) *TRAP* and *Cathepsin K* expression in WT, SC, R1, R2, P1 and P2 RAW264.7 osteoclastic cells after four and eight days osteoclastic induction. Data represented as mean and SD of three experiments of each group. (**m**) Resorptive activity assay in WT, SC, R1 and R2 RAW264.7 cells after seven days of osteoclastic induction. Resorptive areas are highlighted in red at 10× magnification. *P < 0.05 compared to *Ppia*^+/+^ or WT cells.

We next sought to confirm our findings in osteoblastic and osteoclastic cell types *in vitro*. First, primary osteoblasts were isolated from *Ppia*^+/+^ and *Ppia*^−/−^ newborn mouse calvaria (Fig. 3a–c). We confirmed loss of CypA by Western blot (Fig. 3a). Osteogenic markers were reduced among *Ppia*^−/−^ osteoblasts, including *ALP (alkaline phosphatase), Ocn* and *Runx2 (runt-related transcription factor 2)* (Fig. 3b). ALP and Alizarin red staining (markers of early and late osteogenic differentiation) were also reduced (Fig. 3c).

*CypA* silencing or forced overexpression was next performed in MC3T3-E1 osteoblastic cells. Two clones for *CypA* silencing are referred to as M1 and M3, while two clones for overexpression are designated P1 and P2. Western blot confirmed the efficacy of *CypA* silencing and overexpression (Fig. 3d). Next, examination of osteogenic genes was examined (Fig. 3e). Across all markers, *CypA* underexpression led to a reduction in osteogenic gene expression. Conversely, *CypA* overexpression led to an increase in osteogenic markers. ALP and Alizarin red staining confirmed our previous gene expression findings (Fig. 3f).

Next, parallel experiments were performed in osteoclastic cells (Fig. 3g–l). Primary osteoclasts were isolated from the bone marrow of three week old mice (Fig. 3g–i). Western blot confirmed loss of CypA (Fig. 3g). Biochemical TRAP (tartrate-resistant acid phosphatase) staining showed an increase in staining among *Ppia*^−/−^ osteoclasts (Fig. 3h). Gene markers of osteoclastic differentiation, *TRAP* and *Cathepsin K,* likewise were increased among *Ppia*^−/−^ osteoclasts (Fig. 3i, assessed at four and eight days).

Next, CypA silencing or overexpression in RAW294.7 osteoclastic cells was performed. Two clones for CypA silencing are referred to as R1 and R2, while two clones for overexpression are designated P1 and P2. Western blot confirmed the efficacy of CypA dysregulation (Fig. 3j). Next, osteoclastic activity was assessed by TRAP staining (Fig. 3k). CypA silencing increased TRAP staining, while forced CypA overexpression reduced TRAP staining. Likewise, CypA silencing led to an increase in osteoclast markers (Fig. 3l). Conversely, CypA overexpression led to a reduction in osteoclast markers. Finally, CypA knockdown resulted in increased resorptive activity (Fig. 3m).

In summary, CypA had inverse effects on osteoblastic and osteoclastic activity. CypA enhanced osteoblastic differentiation while inhibiting osteoclastic activity. These findings were in agreement with the skeletal phenotype of the *Ppia*^−/−^ mouse.

### Downstream molecular effects of CypA knockdown in osteoblastic and osteoclastic cells

**Figure 4:**
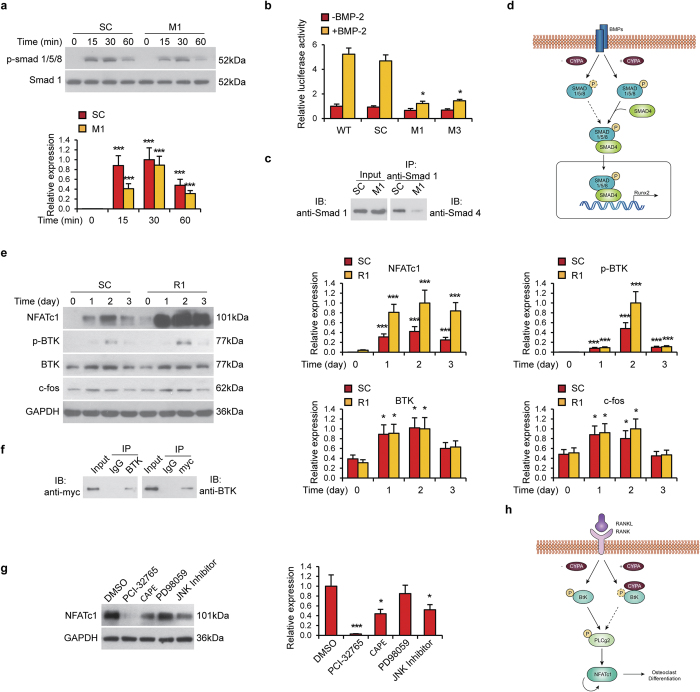
CypA affects mouse osteoblast and osteoclast via different signaling pathways. (**a**–**d**) CypA regulation of BMP-2 induced Smad phosphorylation in osteoblastic cells. (**a**) SC and M1 MC3T3-E1 osteoblastic cells were treated with BMP-2. Cells were harvested for western blot of p-Smad1/5/8 expression at various timepoints. Smad1 was used as a loading control. (**b**) Id1 Luciferase activity of WT, SC, M1 and M2 cells in the presence of BMP2. Data represented as mean and SEM from three independent experiments. *P < 0.05 by one way ANOVA analysis, compared to WT cells in the same group. (**c**) Co-immunoprecipitation between Smad1 and Smad4 in SC and M1 MC3T3-E1 osteoblastic cells. (**d**) Schematic diagram of CypA in osteoblastic differentiation. E-H CypA regulation of downstream signaling in osteoclastic cells. (**e**) p-BTK, BTK, c-fos and NFATc1 levels in SC and R1 RAW263.7 osteoclastic cells during osteoclastic induction at various timepoints as assessed by western blot. GAPDH was used as a loading control. (**f**) Myc-tag CypA plasmid was transfected into RAW264.7 cells for two days, follow by co-immunoprecipitation assay between myc-CypA and BTK. (**g**) NFATc1 levels in differentiated RAW264.7 cells (R1 clone) treated with PCI-32765, CAPE, PD98059 or JNK inhibitor for two days. GAPDH was used as a loading control. (**h**) Schematic diagram of CypA in osteoclastic differentiation.

The dual pro-osteogenic, anti-osteoclastic effects of CypA were further examined by interrogation of downstream signaling elements. First, MC3T3-E1 osteoblastic cells were cultured in the presence or absence of BMP-2, with or without CypA silencing) (Fig. 4a–c). CypA silencing resulted in a reduction in the degree and length of BMP-2 induced Smad1/5/8 phosphorylation (Fig. 4a). Similar findings were confirmed with Id1 (Inhibitor of differentiation)-luciferase activity (Fig. 4b). Next, co-immunoprecipitation assays were performed for Smad1 and Smad4 in the context of CypA silencing (Fig. 4c). Loss of CypA led to a reduction in Smad4/Smad1 binding (Fig. 4c). Thus, CypA expression is required for BMP-2 induced Smad1/5/8 phosphorylation, which subsequently recruits Smad4 into the complex to exert transcriptional activator to regulate Runx2 and results in Smad1/Smad4 binding (see Fig. 4d for a schematic representation).

Next, the downstream effects of CypA loss were examined in RAW297.4 osteoclastic cells (Fig. 4e–h). First, phospho-BTK, c-fos and NFATc1 expression were examined across early osteoclastic induction (days 0–3) with CypA silencing (Fig. 4e). CypA knockdown resulted in an increase in BTK phosphorylation and NFATc1 expression (Fig. 4e). Next, co-immunoprecipitation assays were performed to evaluate CypA (myc-tagged CypA) binding to BTK (Fig. 4f). Results suggested that CypA interacts with BTK and hinders BTK phosphorylation. Finally, NFATc1 expression among the R1 clone was examined in the context of multiple signaling inhibitors, including the BTK inhibitor PCI-32765 (Fig. 4g). Results showed that PCI-32765 substantially reduced NFATc1 expression, while the NF-κB inhibitor CAPE (Caffeic acid phenethyl ester) or JNK inhibitor partially reduced NFATc1 expression. Thus, loss of CypA resulted in BTK activation and integrated with TRAF6 and/or c-fos signaling to induce NFATc1 expression in osteoclastic cells (see Fig. 4h for a schematic representation). Our results are in agreement with the previously reported crucial roles of the tyrosine kinases Btk and Tec in RANKL-induced osteoclastogenesis, based on the genetic evidence obtained from *Tec*^−/−^, *Btk*^−/−^ mice^15^.

## Discussion

In summary, we have identified a novel dual function of CypA in the regulation of osteoblastic and osteoclastic activity. Although further study is warranted, CypA appears to differentially regulate signaling pathways in osteoblasts and osteoclasts. In osteoblasts, CypA is required for BMP-2 induced phosphorylation of Smad1/5/8. In osteoclasts, loss of CypA activates BtK and induces NFATc1 expression via TRAF6 and/or c-fos signaling to induce NFATc1 expression.

Although the *Ppia*^−/−^ mouse shows a low BMD skeletal phenotype throughout its life, the manifestations are most severe during early development. Unfortunately, a skeletal analysis at later ages is not possible due to postnatal lethality. One potential explanation for these age-dependent effects may be attributed to the activity of other isomerases which could compensate for the loss of function of CypA in older age. This result is similar to the previous report that Phd3 (prolyl hydroxylase 3) compensates for the loss of Phd2 (prolyl hydroxylase 2) in the vertebrae^16^. Further studies are needed to investigate if a similar compensation is observed in the *Ppia*^−/−^ mouse skeleton.

One of the surprising findings of the current study was the anti-osteoclastic effect of CypA. Our *in vitro* findings suggest that CypA has direct effects on osteoclasts, although secondary paracrine changes produced by CypA deficiency within the bone marrow milieu cannot be excluded. The manifestations of CypA deficiency are distinct from the results of Pin1 (peptidyl-prolyl cis-trans isomerase NIMA-interacting 1) deficiency^17^. Pin1, another peptidyl-prolyl isomerase, was recently reported to promote osteoblastic activity^17^. For example, *Pin1* deficient mice exhibit a reduced BMD skeletal phenotype accompanied by decreased osteoblastic activity and osteoblast-associated BMP signaling. Moreover, Pin1 appears to regulate BMP signaling, potentially via interaction with Smad5^17^. Despite these similarities between CypA and Pin1, their effects on osteoclasts are distinct. While CypA negatively regulates both the number and activity of osteoclasts, Pin1 deficiency seems to have no effect^17^. These similarities and differences suggest that CypA and Pin1 exert distinct molecular mechanisms to ultimately affect bone anabolism and bone resorption.

Although CypA clearly exerts important skeletal effects, the precise cell type most affected by CypA deficiency is unknown. We previously reported on the effects of CypA on cartilage, showing that CypA acts on committed chondrocytes primarily via regulation of NF-kB and the transcription factor Sox9^6^. In the present study it is clear that CypA acts on committed osteoblasts via regulation of Smad1/5/8 phosphorylation, subsequent Smad4 recruitment, and regulation of Runx2. The potent effects of CypA on cells of the osteochondral lineage suggest the possibility that CypA exerts its effects on mesenchymal stem cells and their progeny. Overall, the relative importance of CypA on differentiated versus undifferentiated osteochondral cells is an interesting topic of future investigation.

The clinical relevance of peptidyl-prolyl isomerase regulation largely comes from studies examining the effects of CsA (cyclosporin A) on bone. CsA has been recognized to have inhibitory effects on PPIase activity^18^,^19^. While not entirely agreed upon, most investigators have reported that CsA has detrimental effects on bone metabolism^20^,^21^,^22^. For example, high dose CsA treatment has catabolic effects resulting in reduced BMD in mice *in vivo*^20^. These studies were further confirmed by Chen *et al*., who reported that CsA treatment of rats resulted in reduced BMD, reduced biomechanical strength, and increased rate of bone fracture^21^. However, investigators have found that effects of CsA may be bimodal. For example, Yeo *et al*. found that while high dose CsA has catabolic effects, low dose CsA appears to have anabolic effects in mice^20^. Similarly, CsA was reported to have anabolic effects in rat alveolar bone formation^23^. Of note, however, upregulation of CypA has been associated with alveolar bone destruction in an experimental model of periodontitis^24^. Thus, it is important to consider the role of PPIase activity in not only the bone, but also the surrounding soft tissues. In summary, these findings support the concept that inhibition of PPIase activity is detrimental to bone health, whereas promotion of PPIase activity may have anabolic effects.

Of course, CypA is not alone in the dual regulation of osteoblastic and osteoclastic activity. Denosumab, a human monoclonal antibody to RANK ligand, is a newer anabolic agent that is administered by subcutaneous injections at 6-month intervals^25^. Denosumab has been shown to inhibit osteoclast activity, decrease bone resorption, and increase bone density. An ongoing Phase II clinical trial (NCT02049866) is examining the use of Denosumab for the prevention of post-teriparatide bone loss in premenopausal women^26^. Anti-Sclerostin antibody (also known as Romosozumab) is another dual anabolic, anti-osteoclastic treatment. In Phase I and II trials, anti-Sclerostin antibody increased bone density of both healthy men and postmenopausal women. Phase III trials are expected to end by late 2015^27^,^28^. Thus, modulation of CypA activity may represent a novel option to regulate both osteoblast and osteoclast activity in order to combat osteoporotic bone loss.

## Methods

### Animal care

All experiments were performed in accordance with institutional guidelines set by the Chancellor’s Animal Research Committee of the Office for Protection of Research Subjects at the University of California, Los Angeles (UCLA) as well as the UCLA Office of Animal Research Oversight. All experimental protocols were approved by Chancellor’s Animal Research Committee at UCLA. *Ppia*^+/−^ mice were purchased from the Jackson Laboratory and backcrossed with C57BL/6 mice for eight generations prior to the experiments.

### Three-dimensional Micro-computed Tomography Evaluation

Long bones and vertebrae were analyzed from mice at three and 13 weeks of age by microCT (micro-computed tomography), as previously described^6^. Six WT and five KO mice in three weeks of age, and six WT and four KO mice in thirteen weeks of age were processed for microCT analysis. WT and KO mice were processed for microCT analysis. Briefly, whole bodies were imaged using high-resolution microCT (SkyScan 1172, Bruker MicroCT N.V., Kontich, Belgium) at an image resolution of 27.4 μm (39 kV and 246 μA radiation source, using a 0.5 mm aluminum filter). The quantitative and structural parameters follow the nomenclatures described by the American Society for Bone and Mineral Research Nomenclature Committee^29^. Common sites of interest were examined, including the tibia, and individual lumbar vertebral bodies (the three terminal vertebral levels).

The optimal threshold for each developing skeletal site was determined based on the normal bone in WT mice. For both three weeks and 13 weeks old animals, threshold ranges of 60–255 and 80–255 were used for trabecular analysis of tibias, and lumbar vertebrae, respectively, and 110–255 was used for cortical analysis of femurs and tibias. Such threshold ranges were selected to optimally represent the normal structures in WT bones, while including slightly less mineralized woven bone structures in the trabecular threshold, and excluding trabecular structures in the cortical threshold.

### Cell culture and differentiation

Primary mouse bone marrow cells (BMC, 2 × 10^6^ cells/ml) were isolated from tibia and femur of 3-week-old male *Ppia*^+/+^ and *Ppia*^−/−^ mice, as previously described^30^. After 48 hours, floating cells (osteoclast, 5 × 10^5^ cells/ml) were collected and cultured in α-MEM medium with 10% FBS (fetal bovine serum), 50 ng/mL m-CSF (macrophage colony-stimulating factor) and 100 ng/mL RANK-L (receptor activator of nuclear factor kappa-B ligand) (BD Biosciences, San Jose, CA) for osteoclastic differentiation. Primary osteoblasts were isolated from the calvaria of newborn mice, as previously described^31^. Primary osteoblasts were cultured until 60% confluency, then 50 μg/ml ascorbic acid and 10 mM β-glycerophosphate (Sigma-Aldrich, St Louis, MO) were added for osteoblastic differentiation. Pre-osteoblast MC3T3-E1 and pre-osteoclast RAW264.7 murine cell lines were obtained from the ATCC (American Type Culture Collection) and maintained in DMEM medium with 10% FBS, 100 U/ml of penicillin, and 100 μg/ml of streptomycin. For osteoclastic differentiation, cultured cells were replacing with α-MEM medium containing 10% FBS, 50 ng/mL m-CSF and 100 ng/mL RANK-L. For osteogenic differentiation, MC3T3-E1 cells (1 × 10^5^ cells/ml) were cultured in growth medium overnight, then 50 μg/ml ascorbic acid and 10 mM β-glycerophosphate were added into the medium.

### Immunohistochemistry (IHC)

Immunohistochemistry analyses were performed as previously described^6^. Briefly, slides were incubated with anti-CypA (1:100; Santa Cruz, Santa Cruz, CA), anti-OCN (1:100; Abcam, Cambridge, MA) or anti-Cathepsin K (1:100, Abcam) overnight at 4°C, after blocking with 1% BSA. The next day, sections were incubated with biotinylated goat anti-rabbit secondary antibody (Santa Cruz) for 1 hour at room temperature. VECTSTAIN Elite ABC complex (Vector lab, Burlingame, CA) incubation was used for primary antibody detection. Negative control sections were incubated in PBS (phosphate-buffered saline) instead of primary antibody.

### Co-immunoprecipitation

Co-immunoprecipitation (Co-IP) was performed as previously described^14^. Briefly, 800 μg of cell lysate was incubated with anti-myc or BTK antibodies at 4 °C overnight in a clinical rotor. Protein A/G PLUS-agarose beads (Santa Cruz) were then added to the mixture and rotated for 3 hours at 4 °C. The tubes were briefly centrifuged at 1000 g at 4 °C for 30 seconds to precipitate the beads bound to the Ag-Ab complex, which was subjected to Western blot analysis probing with anti-myc or anti-BTK antibody.

### Knockdown and overexpression of CypA in MC3T3 and RAW264.7 cells

Generation of stable knockdown cell lines was performed as previously described^6^,^14^,^32^. To knockdown *CypA* in MC3T3 and RAW264.1 cells, the cells were first transfected with the pSilencer-CypARNAi vector^12^ (synthesized by Invitrogen, Carlsbad, CA, USA) using lipofectamine 2000 (Invitrogen) for 48 hours, followed by screening in 500 mg/ml of G418 (Sigma-Aldrich) for 48 hours. Twelve single clones of each cell line were selected and maintained in DMEM (Dulbecco’s Modified Eagle’s Medium) with 10% FBS and 200 mg/ml of G418 for two weeks. For CypA overexpression, cells were transfected with CypA-pcDNA3.1 plasmid^12^ using lipofectamine 2000 for 48 hours. Western blot was performed to determine the efficiency of knockdown or overexpression. Based on their efficiency, M1 and M3 clones (MC3T3-E1 cells), and R1 and R2 single clones (RAW264.7) were selected and compared to SC (scrambled clones) as well as wild-type parental cells.

### Quantitative Real-time PCR (qRT-PCR)

Quantitative real-time PCR was performed as described^6^. Total mRNAs (messenger RNA) were extracted with Trizol (Invitrogen) according to the manufacturer’s instructions. First strand cDNA (complementary DNA) synthesis and amplification was performed using Superscript® III Reverse Transcription System (Invitrogen). qRT-PCR reactions were performed in a total volume of 25 μl containing 2 μM primers and 12.5 μl of the Power SYRB green PCR master mix in triplicates (Applied Biosystems, Foster City, CA, USA). Statistical analysis of qRT-PCR data was performed using 2^−ΔΔCT^ values. The primers used for RT-PCR were: *ALP (NM_007431.3)* forward 5′-CACAATATCAAGGATATCGA-CGTGA-3′, reverse 5′-ACATCAGTTCTGTTCTTCGGGTACA-3′; *OCN (NM_001305448)* forward 5′-GGGCAATAAGGTAGTGAACAG-3′, reverse 5′-GCAGCACAGGTCC-TAAATAGT-3′; *Runx2 (NM_001145920)* forward 5′-TTACCTACACCCCGCCAGTC-3′, reverse 5′-TGCTGGTCTGGAAGGGTCC-3′; *TRAP (NM_001102404)* forward 5′-ACACAGT-GATGCTGTGT-GGCAACTC-3′, reverse 5′-CCAGAGGCTTCCACATATATGATGG-3′; *Cathepsin K (NM_007802)* forward 5′-GTTGTATGTATAACGCCACGGC-3′, reverse 5′-CTTTCTCGTTCCCCACAGGA-3′; *GAPDH (NM_001289726)* forward 5′-AAATGGTGAA-GGTCGGTGTG-3′, reverse 5′-TGAAGGGGTCGTT-GATGG-3′. QRT-PCR experiments were performed after seven days osteogenic differentiation, or after four and eight days of osteoclastic differentiation.

### Alkaline phosphatase (ALP) staining and mineralization assay

Primary mouse osteoblasts or MC3T3-E1 cells were seeded in 24-well plates and induced for osteogenic differentiation for seven days. Cells were rinsed with PBS, fixed in 4% paraformaldehyde (PFA), rinsed with PBS, and then ALP staining was performed using a solution (pH 8.2–8.5) containing 0.5 mg/ml naphthol AS-BI phosphate (Sigma-Aldrich), 0.5 mg/ml Fast Blue (Sigma-Aldrich), 10% N,N-dimethylformamide (Sigma-Aldrich), 0.5% MgCl_2_ in 0.1 M Tris for 10 min at room temperature in the dark.

Mineralization assay was performed as previously described^6^. Briefly, primary mouse osteoblasts or MC3T3-E1 cells were cultured in 24-well plates and osteogenic differentiation was performed for three weeks. Cells were stained with Alizarin Red S solution (PH 4.2, Sigma-Aldrich) for 10 min at room temperature and washed three times with water.

### TRAP (Tartrate-resistant acid phosphatase) staining

Primary mouse osteoclasts and RAW264.7 cells were seeded in 24-well plates and differentiated for seven days. After induction of differentiation, cells were washed with phosphate buffered saline (PBS) and fixed in paraformaldehyde 4% in PBS for 10 min, following by incubating for 20 min at 37 °C in the TRAP staining solution (Sigma-Aldrich) according to the manufacturer’s instructions.

### Resorption assay and assessments

Bone resorption assay was performed as previously described^33^. Briefly, RAW 264.7 cells (5 × 10^3^ cells per well) were seeded in Corning Osteo-Assay Surface 96-well plates (Corning Life Sciences) in 500 μl of complete α-MEM containing 10% FBS, 10^−7^ M PGE2, 10 ng/ml M-CSF and 10 ng/ml recombinant RANKL. Cells were incubated at 37 °C in a humidified 5% CO_2_ incubator and medium was changed every day. The cells were incubated for seven days prior to processing for Von Kossa staining.

To perform Von Kossa staining, plates were stripped with 10% bleach for 5 min to remove cells, rinsed with distilled water, and air-dried until further use. Plates were treated with 500 μl/well 1% (w/v) silver nitrate solution and placed in front of a 75 watt light bulb for 1 hour. Wells were then washed for 5 min in distilled water. Un-reacted silver was removed with 5% (w/v) sodium thiosulfate (500 μl/well, 5 min). The plates were then washed and air dried prior to imaging. All microplate wells were imaged with six fields/well (10×). Percentage of the resorbed surface was carried out with NIH ImageJ software.

### Statistical analysis

All values are expressed as mean ± SEM (standard error of the mean), unless otherwise stated. One way ANOVA analysis of variance and Student’s *t*-test was analyzed using SPSS 20.0 for windows. *P* values of less than 0.05 were considered to be significant. All experiments were performed in triplicate and repeated at least twice.

## Additional Information

**How to cite this article**: Guo, M. *et al*. Cyclophilin A (CypA) Plays Dual Roles in Regulation of Bone Anabolism and Resorption. *Sci. Rep.* **6**, 22378; doi: 10.1038/srep22378 (2016).

## References

[b1] Becker D. J., Kilgore M. L. & Morrisey M. A. The societal burden of osteoporosis. Curr. Rheumatol. Rep. 12, 186–191 (2010). 20425518 10.1007/s11926-010-0097-y

[b2] Rasmussen H. & Bordier P. The cellular basis of metabolic bone disease. N. Engl. J. Med. 289, 25–32 (1973). 4575395 10.1056/NEJM197307052890107

[b3] Mellis D. J., Itzstein C., Helfrich M. H. & Crockett J. C. The skeleton: a multi-functional complex organ: the role of key signalling pathways in osteoclast differentiation and in bone resorption. J. Endocrinol. 211, 131–143 (2011). 21903860 10.1530/JOE-11-0212

[b4] Canalis E., Giustina A. & Bilezikian J. P. Mechanisms of anabolic therapies for osteoporosis. N. Engl. J. Med. 357, 905–916 (2007). 17761594 10.1056/NEJMra067395

[b5] Lu K. P., Finn G., Lee T. H. & Nicholson L. K. Prolyl cis-trans isomerization as a molecular timer. Nat. Chem. Biol. 3, 619–629 (2007). 17876319 10.1038/nchembio.2007.35

[b6] Guo M. . A novel role of Cyclophilin A in regulation of chondrogenic commitment and endochondral ossification. Mol. Cell. Biol. (2015). 10.1128/MCB.01414-14 PMC4438250 25870110

[b7] Hong F. . Cyclosporin A blocks muscle differentiation by inducing oxidative stress and inhibiting the peptidyl-prolyl-cis-trans isomerase activity of cyclophilin A: cyclophilin A protects myoblasts from cyclosporin A-induced cytotoxicity. FASEB J. 16, 1633–1635 (2002). 12207006 10.1096/fj.02-0060fje

[b8] Satoh K. . Cyclophilin A promotes cardiac hypertrophy in apolipoprotein E-deficient mice. Arterioscler. Thromb. Vasc. Biol. 31, 1116–1123 (2011). 21330604 10.1161/ATVBAHA.110.214601 PMC3085960

[b9] Tian-Tian Z., Jun-Feng Z. & Heng G. Functions of cyclophilin A in atherosclerosis. Exp. Clin. Cardiol. 18, e118–124 (2013). 23940449 PMC3718612

[b10] Colgan J., Asmal M., Yu B. & Luban J. Cyclophilin A-deficient mice are resistant to immunosuppression by cyclosporine. J. Immunol. 174, 6030–6038 (2005). 15879096 10.4049/jimmunol.174.10.6030

[b11] Colgan J. . Cyclophilin A regulates TCR signal strength in CD4^+^ T cells via a proline-directed conformational switch in Itk. Immunity 21, 189–201 (2004). 15308100 10.1016/j.immuni.2004.07.005

[b12] Song J., Lu Y. C., Yokoyama K., Rossi J. & Chiu R. Cyclophilin A is required for retinoic acid-induced neuronal differentiation in p19 cells. J. Biol. Chem. 279, 24414–24419 (2004). 15047706 10.1074/jbc.M311406200

[b13] Bauer K. . Cyclophilins contribute to Stat3 signaling and survival of multiple myeloma cells. Oncogene 28, 2784–2795 (2009). 19503092 10.1038/onc.2009.142

[b14] Sun S. . Cyclophilin A (CypA) Interacts with NF-kappaB Subunit, p65/RelA, and Contributes to NF-kappaB Activation Signaling. PLoS One 9, e96211 (2014). 25119989 10.1371/journal.pone.0096211 PMC4130471

[b15] Shinohara M. . Tyrosine kinases Btk and Tec regulate osteoclast differentiation by linking RANK and ITAM signals. Cell 132, 794–806 (2008). 18329366 10.1016/j.cell.2007.12.037

[b16] Cheng S., Xing W., Pourteymoor S. & Mohan S. Conditional disruption of the prolyl hydroxylase domain-containing protein 2 (Phd2) gene defines its key role in skeletal development. J. Bone Miner. Res. 29, 2276–2286 (2014). 24753072 10.1002/jbmr.2258

[b17] Shen Z. J. . Pin1 null mice exhibit low bone mass and attenuation of BMP signaling. PloS one 8, e63565 (2013). 23675491 10.1371/journal.pone.0063565 PMC3651169

[b18] Takahashi N., Hayano T. & Suzuki M. Peptidyl-prolyl cis-trans isomerase is the cyclosporin A-binding protein cyclophilin. Nature 337, 473–475 (1989). 2644542 10.1038/337473a0

[b19] Handschumacher R. E., Harding M. W., Rice J., Drugge R. J. & Speicher D. W. Cyclophilin: a specific cytosolic binding protein for cyclosporin A. Science 226, 544–547 (1984). 6238408 10.1126/science.6238408

[b20] Yeo H., Beck L. H., McDonald J. M. & Zayzafoon M. Cyclosporin A elicits dose-dependent biphasic effects on osteoblast differentiation and bone formation. Bone 40, 1502–1516 (2007). 17392048 10.1016/j.bone.2007.02.017 PMC1974856

[b21] Chen Y., Zheng X., Zou R. & Wang J. Effects of cyclosporin-a on rat skeletal biomechanical properties. BMC Musculoskeletal Disord. 12, 240 (2011). 10.1186/1471-2474-12-240 PMC3213210 22024110

[b22] Movsowitz C. . Combined treatment with cyclosporin A and cortisone acetate minimizes the adverse bone effects of either agent alone. J. Orthop. Res. 8, 635–641 (1990). 2388102 10.1002/jor.1100080503

[b23] Cetinkaya B. O., Acikgoz G., Keles G. C., Ayas B. & Korkmaz A. The effect of cyclosporin A on alveolar bone in rats subjected to experimental periodontal disease. Toxicol. Pathol. 34, 716–722 (2006). 17162529 10.1080/01926230600826269

[b24] Liu L., Li C., Cai C., Xiang J. & Cao Z. Cyclophilin A (CypA) is associated with the inflammatory infiltration and alveolar bone destruction in an experimental periodontitis. Biochem. Biophys. Res. Commun. 391, 1000–1006 (2010). 19968957 10.1016/j.bbrc.2009.12.005

[b25] Lewiecki E. M. Denosumab: an investigational drug for the management of postmenopausal osteoporosis. Biol. Targets. Ther. 2, 645–653 (2008). 10.2147/btt.s2082 PMC2727882 19707445

[b26] Bragdon B. . Bone morphogenetic proteins: a critical review. Cell Signal 23, 609–620 (2011). 20959140 10.1016/j.cellsig.2010.10.003

[b27] Amgen. *UCB and Amgen Initiate Sclerostin Antibody Phase 3 Program in Patients With Postmenopausal Osteoporosis* (2012). Available at: http://www.amgen.com/media/media_pr_detail.jsp?releaseID=1679935. (Accessed 29^th^ Sept. 2015).

[b28] Nohe A. . The mode of bone morphogenetic protein (BMP) receptor oligomerization determines different BMP-2 signaling pathways. J. Biol. Chem. 277, 5330–5338 (2002). 11714695 10.1074/jbc.M102750200

[b29] Parfitt A. M. . Bone histomorphometry: standardization of nomenclature, symbols, and units. Report of the ASBMR Histomorphometry Nomenclature Committee. J. Bone Miner. Res. 2, 595–610 (1987). 3455637 10.1002/jbmr.5650020617

[b30] Takahashi N., Udagawa N., Tanaka S. & Suda T. Generating murine osteoclasts from bone marrow. Methods Mol. Med. 80, 129–144 (2003). 12728715 10.1385/1-59259-366-6:129

[b31] Takahashi N. . Osteoblastic cells are involved in osteoclast formation. Endocrinology 123, 2600–2602 (1988). 2844518 10.1210/endo-123-5-2600

[b32] Calhoun C. C., Lu Y. C., Song J. & Chiu R. Knockdown endogenous CypA with siRNA in U2OS cells results in disruption of F-actin structure and alters tumor phenotype. Mol.Cell Biochem. 320, 35–43 (2009). 18704644 10.1007/s11010-008-9896-0

[b33] Tevlin R. . Osteoclast derivation from mouse bone marrow. J. Vis. Exp. e52056 (2014). 25407120 10.3791/52056 PMC4353410

